# Alterations in the Brain Structure and Functional Connectivity in Aquaporin-4 Antibody-Positive Neuromyelitis Optica Spectrum Disorder

**DOI:** 10.3389/fnins.2019.01362

**Published:** 2020-01-14

**Authors:** Jueyue Yan, Yu Wang, Hanpei Miao, William Robert Kwapong, Yi Lu, Qingkai Ma, Wei Chen, Yunhai Tu, Xiaozheng Liu

**Affiliations:** ^1^Department of Neurology, The Second Affiliated Hospital & Yuying Children’s Hospital, Wenzhou Medical University, Wenzhou, China; ^2^China-USA Neuroimaging Research Institute, Department of Radiology, The Second Affiliated Hospital & Yuying Children’s Hospital, Wenzhou Medical University, Wenzhou, China; ^3^School of Ophthalmology and Optometry, Wenzhou Medical University, Wenzhou, China; ^4^Department of Opthalmology, The First Affiliated Hospital of Anhui Medical University, Hefei, China; ^5^Department of Psychiatry, Sir Run Run Shaw Hospital, Collaborative Innovation Center for Brain Science, Zhejiang University School of Medicine, Hangzhou, China

**Keywords:** gray matter volume, functional connectivity, neuromyelitis optica spectrum disorder, resting-state functional magnetic resonance imaging, optic neuritis

## Abstract

**Purpose:**

To investigate the mechanisms underlying the gray matter volume (GMV) and functional connectivity (FC) changes in aquaporin-4 antibody-positive neuromyelitis optica spectrum disorder (NMOSD) patients.

**Methods:**

This cross-sectional study consisted of 21 patients with aquaporin-4 antibody-positive NMOSD and 22 age- and sex-matched healthy controls. All participants underwent cerebral magnetic resonance imaging and testing each individual’s visual acuity was done.

**Results:**

Neuromyelitis optica spectrum disorder patients showed significantly reduced GMV in the left calcarine, left thalamus and right lingual gyrus of the NMOSD patients when compared to HC (*P* < 0.05). NMOSD patients showed significantly decreased FC values (*P* < 0.05) in both the left and right calcarine, right lingual gyrus and left thalamus, respectively, when compared to HC. We also observed a positive correlation between the FC values of the left thalamus, bilateral calcarine gyrus and the visual acuity, respectively (*P* < 0.05). Furthermore, a negative association was seen between the duration of the disease, frequency of optic neuritis, and the FC values in the lingual gyrus, bilateral calcarine gyrus, and right lingual gyrus, respectively (*P* < 0.05).

**Conclusion:**

Reduced visual acuity and frequency of optic neuritis are associated with alterations in the GMV and FC in NMOSD. Our current study, which provides imaging evidence on the impairment involved in NMOSD, sheds light on pathophysiological responses of optic neuritis attack on the brain especially on the visual network.

## Introduction

Neuromyelitis Optica spectrum disorders (NMOSD) is a rare autoimmune inflammatory disorder of the central nervous system (CNS) which affects the brain and eye and is characterized by attack(s) of optic neuritis (ON) and myelitis (Jarius et al., 2014). The distinctive clinical features of NMOSD are decreased vision (which may later lead to blindness), pain during eye movement, and visual field defects after attack(s) of optic neuritis (Vabanesi et al., 2019; Wu et al., 2019). Previous reports have stressed on the significantly decreased vision that results from ON in NMOSD; in a study by Wingerchuk et al. (1999), over 50% of NMOSD patients who were relapsing, were almost blind in at least an eye in the US while a study by Kitley et al. (2012) reported of bilateral permanent visual loss in a portion of NMOSD patients in the United Kingdom and Japan. Patients develop monophasic or recurrent optic neuritis, longitudinally extensive transverse myelitis, and brainstem lesions that can lead to substantial disability and even death (Silbermann and Bourdette, 2019).

Improvement in neuroimaging technology has brought forth both the structural and functional data in brains; currently, NMOSD patients with or without ON can be imaged effortlessly. Voxel-based morphometry (VBM) is a whole-brain morphology analysis that compares the voxel-wise, intra-group differences in local brain morphology (Ashburner and Friston, 2000). A neuroimaging report from Chan et al. (2011) showed significant changes that occurred in the microstructural volume of NMOSD patients when compared to healthy controls (HC). A previous study based on VBM has shown significant loss in gray matter volume (such as the thalamus) in patients with NMOSD after ON when compared to healthy controls (Kim et al., 2015). Furthermore, longitudinal studies using VBM on NMOSD showed significant loss of gray matter especially in patients with recurrent ON attack (Kim et al., 2012; Carnero Contentti et al., 2018). Another study also showed that changes in the microstructural of the gray matter affect the visual network of the brain thus affecting the vision of NMOSD patients (Pittock and Lucchinetti, 2016).

Functional magnetic resonance imaging (fMRI) using a bold oxygenation level-dependent (BOLD) signal is one of the most versatile and vital tools to study non-invasive functional activation of the human brain *in vivo*. Functional connectivity (FC) in resting-state functional MRI (RS-MRI) reflects the correlation of the blood-oxygen level-dependent signals in different portions of the brain in a time sequence; seed correlation analysis is one of the common FC analysis used. Both VBM and FC methods have been widely used in NMOSD and multiple sclerosis (MS) (Cruz Gomez et al., 2013) studies; however, understanding the structural and functional significance of ON in the brain of MS disease cascade is limited by the presence of demyelinating lesions in the optic radiation and typically relatively mild visual impairment. In contrast, NMOSD represents a definitive model for the exploration of functional changes in the brain after ON, given the non-appearance of optic radiation injuries in typical cases together with severe damage to the visual system.

Microstructural and functional neuroimaging studies have separately reported the functional and structural alterations associated with NMOSD, yet, the relationship between these microstructural and functional changes remains ambiguous. Benoliel et al. (2017) have shown the association between clinical symptoms and structural and functional cerebral changes in NMOSD but very little is known of the changes that occur in the brain activity and volume after ON attack(s). We hypothesize that there may be changes in the visual cortex after demyelination of the optic nerve in NMOSD patients after ON attack(s).

Thus our current study combined the VBM method with the seed-based resting-state analysis method to explore the visual cortex and the whole brain FC changes in NMOSD after ON attack(s) (1) assessing the differences in gray matter volume (GMV) and FC between NMOSD patients and HC, (2) exploring the association between FC measurements and the clinical information, and (3) investigating the mechanisms underlying the FC changes in the frequency of ON attacks.

## Materials and Methods

### Demographics

Twenty-five patients diagnosed with NMOSD who met the 2015 international consensus diagnostic criteria of NMOSD (Wingerchuk et al., 1999) were enlisted from the outpatient of the Neurology Department, The Second Affiliated Hospital and Yuying Children’s Hospital of Wenzhou Medical University between January 2019 and June 2019. AQP-4 antibody tests were implemented for all patients by the hospital’s specialized laboratory. The inclusion criteria for patients were Chinese ethnicity and anti-AQP4 antibody seropositive. Patients who were MOG antibodies positive were excluded. All patients recruited were relapse-free for 6 months or more and without high dose corticosteroid treatment before MRI investigation. Other inclusion criteria included: 1. Vision loss with or without pain in the eye, 2. Visual field defects associated with damage to the nerve fibers of the eye, 3. Exclusion of other possible diagnoses, such as toxic, genetic, metabolic or invasive optic neuropathy, and 4. Sufficient MR image quality. Four NMOSD patients were excluded after MR imaging due to poor imaging quality. The control group included 22 controls from the hospital workers or interns in the hospital who were age and sex-matched; controls who had no history of neurologic or psychiatric disease and were matched for age and sex. The exclusion criteria were: 1. No neurological or psychiatric illness, 2. No visual system-related diseases, 3. No abnormality on MR imaging. All participants underwent a baseline clinical MRI; none of the participants were found having brainstem syndromes. The Ethics Committee of The Second Affiliated Hospital and Yuying Children’s Hospital of Wenzhou Medical University approved of this study and was in accordance with the Declaration of Helsinki. All participants provided informed consent before enrollment in our study.

### Aquaporin-4 Immunofluorescence Assay

Analysis for serum samples for the presence of AQP-4 antibodies by extracellular live cell-staining immunofluorescence technique using transiently transfected AQP-4 expressing cells as previously reported (Mader et al., 2010; Di Pauli et al., 2011). Screening for the detection of AQP-4 was done at dilutions of 1:20 and 1:40 by specialized clinicians.

### Measurement of Visual Acuity

Early Treatment Diabetic Retinopathy Study chart at a 3.2-m distance was used for the measurement of each participant’s visual acuity. Each participant underwent both monocular and binocular visual acuity evaluation. For patients with very poor vision, fingers were held before the eye or hand movements were done and later transformed as visual acuity (Schulze-Bonsel et al., 2006).

### MRI Data Acquisition

Siemens 3 Tesla Trio Tim MRI scanner equipped with a 32-channel head coil was used to image the brain of each participant. The specifications of the MRI used in our current study have been reported in a previous study (Bai et al., 2019).

### MRI Data Preprocessing

For structural MR images, FSL’s standard VBM processing pipeline was implemented. An optimized VBM approach as reported by Good et al. (2001) was implemented with all processing steps carried out by means of open ware FSL version 4.1.5^1^ (Beckmann et al., 2006). The pre-processed structural scans for all scanning sessions for a given subject were first rigidly aligned to the first scan using FSL non-linear registration tool (FLIRT) and a mean image was created. Next, tissue-type segmentation was carried out on the subject mean image using FSL automated segmentation tool (FAST). The resulting gray-matter partial volume images were then aligned to MNI152 standard space. The resulting images were averaged to create a study-specific template. The brain-extraction and segmentation steps were then repeated on the rigidly aligned structural scans from each scanning session. The segmented native gray matter images were then non-linearly registered to the template using the transformations calculated from the averaged images. The segmented images were then smoothed with an isotropic Gaussian kernel with a sigma of 3 mm.

All fMRI preprocessing was performed using Statistical Parametric Mapping version 8 (SPM8; Welcome Institute of Cognitive Neurology, London) and Data Processing Assistant for rs-fMRI advanced edition (DPARSFA)^2^ in MATLAB software (R2014a) for the resting-state fMRI (rs-fMRI). The first ten time points of each subject were discarded to allow the signal to reach equilibrium and the participants to adapt to the scanning noise. In sequence, slice timing, head motion correction, and spatial normalization to the standard Montreal Neurological Institute (MNI) template with a resampled voxel size of 3 mm × 3 mm × 3 mm. Subjects with head movement less than 2 mm translation in any axis or less than two angular rotations in any axis during fMRI scanning were included. After that, the data were detrended to remove the linear trend of time courses and were band-pass filtered (temporal band-pass filtering is 0.01–0.08 Hz). Finally, global signal, white matter signal, and cerebral-spinal fluid signal were regressed out as covariates.

For each seed region, a voxel-wise FC analysis was performed separately for each ROI. The mean time series from all of the voxels within the ROI was used as the seed reference time series, and the Pearson’s correlation coefficient (between the average time series for that seed and each voxel in the brain) was computed as the strength of FC. For further statistical analysis, the correlation coefficients were transformed to *z*-values using the Fisher r-to-z transformation to improve the normality of the correlation coefficients (Finke et al., 2018). Thus, a map that represented the FC strength and the seed region (in terms of the *z*-values for each subject) was obtained. Four NMOSD patients were excluded due to poor imaging quality.

### FSL Voxel-Based Morphometry (FSL-VBM)

We performed non-parametric statistical analysis using FSL Randomize with 5,000 permutations and threshold-free cluster enhancement (TFCE) option (*P* < 0.05).

### Seed-Based Resting-State Functional Connectivity Analysis

A two-tailed *t*-test was used for comparisons of the FC between the groups. Correction for multiple comparisons was accomplished with 3dClustSim using analysis of Functional NeuroImages (AFNI^3^). *P* < 0.05 was considered statistically significant. All results were viewed on the MNI T1 template and the p or *T*-value scale is shown on the right of the image.

### Statistical Analysis

Statistical analyses for demographics and clinical measures were performed using SPSS version 21. Multiple linear regression was used to assess the association between FC, gray matter changes, visual acuity, and disease duration, respectively. Independent-sample *t*-tests and the Fisher exact test were used where appropriate.

## Results

### Demographics and Clinical Information

Twenty-one patients with NMOSD (mean age 48.0 years) and 22 healthy controls (mean age 47.78) participated in this study. Significant differences (*P* > 0.05) were not seen in age, BMI, and gender. Significant differences (*P* < 0.001) were seen in the disease duration and best corrected visual acuity (BCVA) between the two groups as shown in Table 1.

**TABLE 1:** Demographics and clinical information.

	**NMOSD**	**Control group**	***P***
Eyes	21	22	
Gender	21/0	22/0	
Age, years	48.0 (12.85)	47.78 (11.63)	0.953
BMI	22.34 (2.41)	23.82 (2.68)	
Duration	5.30 (4.50)	–	<0.001
BCVA	0.44 (0.42)	1.11 (0.16)	<0.001
AL	23.43 (1.14)	23.35 (1.13)	0.843
IOP	13.70 (3.51)	12.16 (2.70)	0.117
**Frequency of ON**			
1 time	8(28.6%)		
2 times	13(57.1%)		

*NMOSD, neuromyelitis optica spectrum disorders; BMI, body mass index; BCVA, best corrected visual acuity; AL, axial length; IOP, intra-ocular pressure; ON, optic neuritis.*

### Comparison of Gray Matter Volume Between Neuromyelitis Optica Spectrum Disorder and Healthy Controls

**FIGURE 1:**
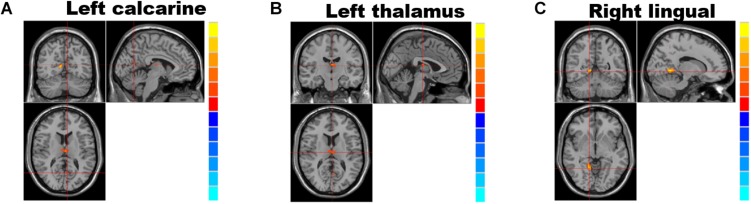
Brain regions showing significantly decreased gray matter volume and group differences (table below) between individuals with neuromyelitis optica spectrum disorder (NMOSD) and controls; from left to right: left calcarine gyrus **(A)**, the left thalamus **(B)**, and right lingual gyrus **(C)**. (*P* < 0.05, threshold-free cluster enhancement (TFCE) correction). The gray matter volumes in the left calcarine gyrus **(A)**, left thalamus **(B)**, and right lingual gyrus **(C)** were significantly reduced (*P* < 0.05) in NMOSD patients when compared to HC.

Analyses of the GMV showed significantly reduced GMV in the left calcarine (*P* = 0.028; Figure 1A and Table 2), left thalamus (*P* = 0.001; Figure 1B and Table 2), and right lingual gyrus (*P* = 0.005; Figure 1C and Table 2) of NMOSD patients when compared to HC. All results of the gray matter volume between NMOSD and HC were shown in the MNI T1 template (Figure 1).

**TABLE 2:** Group differences between individuals with neuromyelitis optica spectrum disorder (NMOSD) and controls.

**Region**	**Brodmann area**	**Cluster size, mm^3^**	**MNI coordinates**		
			***x***	***y***	***z***
R lingual	18	179	14	−54	−4
L thalamus		54	−2	−16	12
L calcarine	17	6	−6	−64	12

*MNI, Montreal Neurological Institute. R lingual, right lingual gyrus. L thalamus, the left thalamus. L calcarine, left calcarine gyrus.*

### Comparison of Functional Connectivity Between Neuromyelitis Optica Spectrum Disorder and Healthy Controls

**FIGURE 2:**
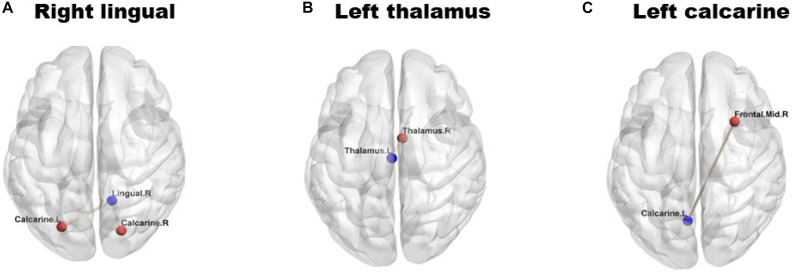
Group differences in functional connectivity (FC) between individuals with neuromyelitis optica spectrum disorder (NMOSD) and controls. The blue dots represent the seed points, and the red dots represent the remaining brain regions displaying differences in FC. The lines represent the functional connections between the seed points and the different brain regions. **(A)** shows the right lingual gyrus; **(B)** shows the left thalamus; **(C)** shows the left calcarine gyrus. The FC values in right lingual gyrus **(A)**, left thalamus **(B)** and left calcarine gyrus **(C)** were significantly reduced (*P* < 0.05) in NMOSD patients when compared to HC.

Analyses of the FC showed significantly decreased FC values in the left [NMOSD = 0.399 (0.250): HC = 0.685 (0.262); *P* = 0.002; Figure 2A] and right [NMOSD = 0.552 (0.230): HC = 0.824 (0.240); *P* = 0.001; Figure 2A] calcarine gyrus, left thalamus [NMOSD = 0.421 (0.192): HC = 0.627 (0.140); *P* < 0.001; Figure 2B], and right lingual gyrus [NMOSD = 0.223 (0.187): HC = 0.359 (0.178); *P* = 0.019; Figure 2C] of NMOSD patients when compared to HC.

### Association Between Aquaporin-4 Levels and Cerebral Parameters

GMV change in the left thalamus (Rho = −0.510, *P* < 0.001) was significantly associated with the AQP-4 levels. FC values in the right lingual gyrus (Rho = −0.385, *P* = 0.011), left (Rho = −0.510, *P* < 0.001) and right (Rho = −0.482, *P* = 0.001) calcarine gyrus, and left thalamus (Rho = −0.538, *P* < 0.001) were significantly associated with AQP-4 levels.

### Association Between Gray Matter Changes and Clinical Information

Gray matter changes in the left thalamus showed significant correlations with frequency (Rho = −0.522, *P* < 0.001), duration (Rho = −0.519, *P* < 0.001), and visual acuity (Rho = 0.444, *P* = 0.003), respectively.

### Association Between Functional Connectivity Changes and Clinical Information

**FIGURE 3:**
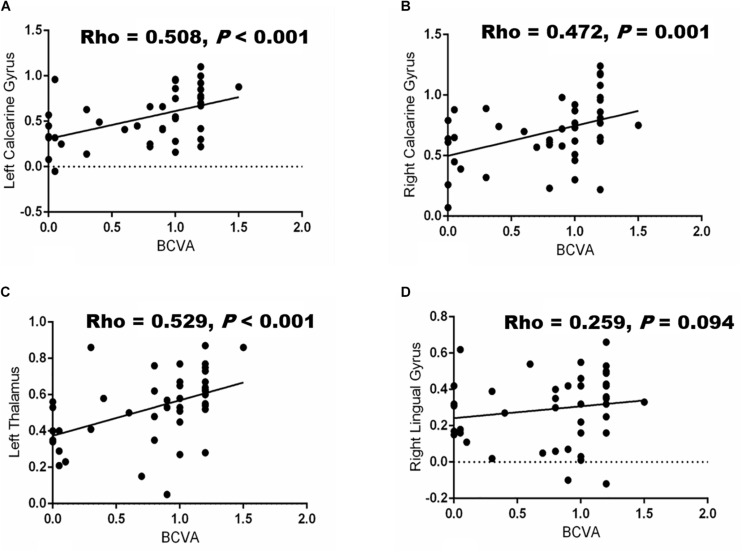
Correlation between BCVA and functional connectivity (FC) values in different brain regions. Significant associations (*P* < 0.05) were shown between BCVA and the changes in FC values of the brain in the left calcarine gyrus **(A)**, right calcarine gyrus **(B)** left thalamus **(C)** and right lingual gyrus **(D)**, respectively.

We observed significant correlations between BCVA and the FC values of the left (Rho = 0.508, *P* < 0.001; Figure 3A) and right (Rho = 0.472, *P* = 0.001; Figure 3B) calcarine gyrus and left thalamus (Rho = 0.529, *P* < 0.001; Figure 3C) but not in right lingual gyrus (Rho = 0.259, *P* = 0.094; Figure 3D), respectively. Furthermore, a negative association was seen between the duration of the disease and the FC values in the right lingual gyrus (Rho = −0.356, *P* = 0.019), left (Rho = −0.516, *P* < 0.001) and right (Rho = −0.450, *P* = 0.002) calcarine gyrus, and left thalamus (Rho = −0.505, *P* = 0.001), respectively. The frequency of optic neuritis was also negatively associated with the FC values in the right lingual gyrus (Rho = −0.325, *P* = 0.033; Table 3), left (Rho = −0.498, *P* = 0.001; Table 3) and right (Rho = −0.565, *P* < 0.001; Table 3) calcarine gyrus, and left thalamus (Rho = −0.650, *P* < 0.001; Table 3), respectively.

**TABLE 3:** Correlation between frequency of ON attack and FC values.

**Regions and BCVA**	**Rho**	***P***
R Lingual	−0.325	0.033
R Calcarine	−0.498	0.001
L Calcarine	−0.565	<0.001
L Thalamus	−0.650	<0.001
ON	−0.751	<0.001

## Discussion

Our current study showed that NMOSD patients with AQP-4 antibody-positive are associated with a pronounced decrease in gray matter and FC in the left thalamus, left calcarine, and right lingual gyrus, respectively, which are constituents of the visual cortex. Furthermore, we showed that changes in the FC were associated with disease duration, visual acuity, and frequency of ON attack, respectively.

A previous report on brain MRI suggested unremarkable changes or non-specific injury in most patients with NMOSD (Kim et al., 2015). On the other hand, the occurrence, clinical manifestations, and changes in cerebral imaging in NMOSD have been reported to be associated with the ethnicity of the patient and the presence of AQP-4 antibody (Cabrera-Gomez et al., 2007, 2008; Pandit et al., 2015). Liu et al. (2015) showed that gray matter damage is a common phenomenon in Chinese patients with NMOSD; however, very little is known of gray matter changes in NMOSD after ON attack(s). Our current study explored a consistent study sample that included AQP-4 antibody-positive NMOSD after ON attack(s) who are Chinese.

AQP-4 antibody has been reported to be expressed in the brain (Badaut et al., 2007); it is the most abundant water channel in the brain (Rash et al., 1998) and controls brain water homeostasis (Nielsen et al., 1997). Amassing reports (Misu et al., 2013; Nishiyama et al., 2016; Yick et al., 2018) have shown that AQP-4 can induce complement independent pathologies. Experimental reports have shown that inflammatory sequela follows the binding of NMO-IgG to AQP-4 (Hinson et al., 2012; Pittock et al., 2013). Kim et al. (2012) suggested that brain MRI abnormalities in AQP-4 autoimmunity are typically localized in the periependymal portions where AQP-4 is highly expressed. Furthermore, reports (Viegas et al., 2009; Samart and Phanthumchinda, 2010) have shown that hypothalamic lesions tend to be more extensive in seropositive AQP-4 NMO patients. Our current report showed that gray matter damage was more severe in the hypothalamus, and a significant association was seen between GMV and AQP-4 levels; thus, we suggest that AQP-4 may contribute to gray matter damage in the brain, which is congruent with aforementioned reports.

Functional MRI is a neuroimaging modality that processes neural activity changes in deoxyhemoglobin levels (blood oxygen level-dependent [BOLD] signal). Thus far, two common methodologies have been suggested: (1) activation fMRI, which evaluates deoxyhemoglobin signal modification during specified tasks, and (2) resting-state fMRI, which associates the synchrony of low-frequency fluctuations of the BOLD signal in various regions while the brain is at rest; accordingly, this technique has been used to determine the FC of neural networks in the brain (Schmidt et al., 2017). Using fMRI, Rocca et al. (2004) showed an irregular form of movement associated with cortical activation in patients with NMOSD that extended beyond the sensorimotor network and involved the visual network of the brain. Previous studies (Lopes et al., 2015; Cai et al., 2017) have also explored visual cortex FC in NMOSD but did not give an account on its association with the clinical data such as their disease duration or frequency of optic neuritis attack; two previous reports (Lopes et al., 2015; Finke et al., 2018) used the activation fMRI approach and showed increased connectivity in NMOSD patients when compared to healthy controls. Cai et al. (2017) used the seed-based approach, similar to our current approach, and showed decreased connectivity in the higher visual cortex. Our current study showed reduced FC values in both the left and right calcarine, right lingual gyrus, and left thalamus; these regions are vital constituents of the visual cortex and visual pathways; thus, we suggest that there is functional damage in the visual network in NMOSD patients.

Our current study showed significantly reduced gray matter volume in the left thalamus, left calcarine, and right lingual gyrus (which are constituents of the primary visual network). Visual impairment, a key clinical manifestation in NMOSD after ON, has been reported to affect the quality of life (Schmidt et al., 2017) since it may lead to blindness (Collongues et al., 2010; Kitley et al., 2012). Thinning of the visual cortex and impaired microstructural integrity restricted to the optic radiation are consistent neuroimaging reports shown in patients with NMOSD (Matthews et al., 2015; Pache et al., 2016). Significant changes in the GMV and FC of the left thalamus suggest that the thalamus, which has been reported to be the central hub in the brain (Usrey and Alitto, 2015), plays a vital role in the frequency of ON attack in NMOSD. The thalamus is an important relay of motor and sensory information to and from the cerebral cortex; additionally, projections to the primary visual cortex are *via* the thalamus, emphasizing its importance in the visual network (Saalmann and Kastner, 2011). A previous report (Tona et al., 2014) on the structural and functional thalamic changes with MS using RS-MRI suggested that increased connectivity in the thalamus could reflect the same disease-associated functional change with demyelination disease. The decreased connectivity in the constituents of the primary visual cortex and the thalamus suggests that the observed changes lead to impoverished visual input due to a large portion (thalamus and other constituents of the visual cortex) of the primary visual cortex being affected. A large portion of the primary visual network with decreased FC suggests that functional reorganization may not be able to compensate the functional damage in the intermittent ON attack in NMOSD patients.

Our current study also showed a high correlation between BCVA and the FC values of the left thalamus and bilateral calcarine gyrus, depicting that changes in the visual network connectivity could be driven by reduced visual input. As such, decreased FC could reflect an attempted compensatory or non-compensatory mechanism of network reorganization in response to the impaired vision in NMOSD. A previous report (Filippi et al., 2013) on MS suggested that increased FC can compensate for structural damage in the brain. On the other hand, the relationship between the changes in the constituents of visual cortex FC and decreased visual acuity could be symptomatic of maladaptive process (Schoonheim et al., 2015). In our current study, we suggest that poor visual input might cause a comparable loss of diversity in visual network connectivity patterns in NMOSD, resulting in an unreliable FC change. Longitudinal studies are needed to validate our hypothesis.

A significant correlation was seen between the FC values and the frequency of ON attack. A previous report (Huang et al., 2018) has shown that recurrent attacks of ON lead to both structural changes and functional alterations of connectivity in the visual network. Our current report echoes what has been previously reported by suggesting that the higher the frequency of ON, the more decreased the FC values will be.

There are some limitations in our current study. First, this study is a cross-sectional study which, although it showed a strong association between FC and impaired visual acuity and disease duration, does not allow the delineation of the underlying causal association. Secondly, the study had a relatively small sample size with limited functional visual measures such as the Snellen chart for visual acuity measurement. The incidence of NMOSD is relatively low (0.5/100000–10/100000) (Cabrera-Gomez et al., 2009; Asgari et al., 2011; Mealy et al., 2012; Flanagan et al., 2016) in China and Asia as a whole when compared to the western world. Wenzhou is a third-class town in China, and most patients decide to visit big cities for treatment which is one of the reasons why we presented with small sample size. Furthermore, our inclusion criteria were quite strict which prohibited the inclusion of four NMOSD patients because they could not meet our inclusion criteria. Our study enrolled 25 NMOSD patients, and 21 were used in our data analyses. Although our sample size is relatively small, our results showed some significant statistical differences when both groups were compared (NMOSD patients and healthy controls). We are currently doing a follow-up on our current study and cooperating with bigger hospitals in larger cities. Further studies with a larger sample size and comprehensive clinical information will be needed to elucidate the associations between the FC measurement and clinical parameters in NMOSD.

## Conclusion

In conclusion, our current study found that NMOSD patients with a history of ON attacks have reduced FC and GMV when compared to healthy controls, and these changes in the brain are associated with their disease duration and frequency of ON attack. Our current study sheds light on the neural mechanism of ON in NMOSD and longitudinal studies with larger sample sizes are needed to validate our hypotheses.

## Data Availability Statement

All datasets generated for this study are included in the article/supplementary material.

## Ethics Statement

The studies involving human participants were reviewed and approved by the Ethics Committee of The Second Affiliated Hospital and Yuying Children’s Hospital of Wenzhou Medical University. The patients/participants provided their written informed consent to participate in this study.

## Author Contributions

All authors listed have made a substantial, direct and intellectual contribution to the work, and approved it for publication.

## Conflict of Interest

The authors declare that the research was conducted in the absence of any commercial or financial relationships that could be construed as a potential conflict of interest.

## Footnotes

1 http://www.fmrib.ox.ac.uk/fsl/

2 http://www.restfmri.net

3 http://afni.nimh.nih.gov/afni/
